# Carbohydrate vs protein supplementation for recovery of neuromuscular function following prolonged load carriage

**DOI:** 10.1186/1550-2783-7-2

**Published:** 2010-01-12

**Authors:** Sam D Blacker, Neil C Williams, Joanne L Fallowfield, James LJ Bilzon, Mark ET Willems

**Affiliations:** 1 University of Chichester, Faculty of Sport, Education and Social Sciences, West Sussex, UK; 2 Institute of Naval Medicine, Gosport, Hampshire, UK; 3 University of Bath, School for Health, Bath, UK

## Abstract

**Background:**

This study examined the effect of carbohydrate and whey protein supplements on recovery of neuromuscular function after prolonged load carriage.

**Methods:**

Ten male participants (body mass: 81.5 ± 10.5 kg, age: 28 ± 9 years,  O_2_max: 55.0 ± 5.5 ml·kg^-1^·min^-1^) completed three treadmill walking tests (2 hr, 6.5 km·h^-1^), carrying a 25 kg backpack consuming 500 ml of either: (1) Placebo (flavoured water) [PLA], (2) 6.4% Carbohydrate Solution [CHO] or (3) 7.0% Whey Protein Solution [PRO]. For three days after load carriage, participants consumed two 500 ml supplement boluses. Muscle performance was measured before and at 0, 24, 48 and 72 h after load carriage, during voluntary and electrically stimulated contractions.

**Results:**

Isometric knee extension force decreased immediately after load carriage with no difference between conditions. During recovery, isometric force returned to pre-exercise values at 48 h for CHO and PRO but at 72 h for PLA. Voluntary activation decreased immediately after load carriage and returned to pre-exercise values at 24 h in all conditions (*P *= 0.086). During recovery, there were no differences between conditions for the change in isokinetic peak torque. Following reductions immediately after load carriage, knee extensor and flexor peak torque (60°·s^-1^) recovered to pre-exercise values at 72 h. Trunk extensor and flexor peak torque (15°·s^-1^) recovered to pre-exercise values at 24 h (*P *= 0.091) and 48 h (*P *= 0.177), respectively.

**Conclusion:**

Recovery of neuromuscular function after prolonged load carriage is improved with either carbohydrate or whey protein supplementation for isometric contractions but not for isokinetic contractions.

## Introduction

Load carriage (i.e. transporting loads in backpacks) is a common endurance exercise in occupational settings (e.g. military services) that causes neuromuscular impairment of the shoulders, trunk and lower limbs [1] and muscle soreness [2]. In the military, fast recovery of muscle function in the days after load carriage is important.

Dietary supplements improve performance during exercise and may aid recovery with their use documented in occupational groups [3]. Interestingly, a reduction in injury rates was observed when 10 g of a protein supplement was provided after exercise compared to a non-protein control during 54 day military basic training course (containing bouts of load carriage) [3].

Recent studies have investigated the effects of protein supplementation on recovery of muscle function after endurance exercise [4] and eccentric exercise [5]. Moreover, supplements with whey protein provide a relatively high proportion of essential amino acids that have a similar amino acid composition to human skeletal muscle [6]. Its benefits have been reported after resistance exercise [7], but as far as we know, the effects of whey protein on recovery of muscle function after load carriage has not been investigated. Ingestion of protein during and after exercise results in a positive protein balance as amino acids are provided for protein synthesis and their presence may also attenuate protein breakdown, potentially influencing recovery of muscle function (e.g. [8]). Combined protein and carbohydrate supplements and carbohydrate only did not enhance recovery of maximal strength of knee extensors from short duration (i.e. 30 min) of eccentric exercise (i.e. downhill running [9]). However, carbohydrates are known to improve endurance exercise performance and enhance recovery with improved subsequent exercise performance [10]. However, the effect of carbohydrate supplementation on recovery of the force producing capability of muscle groups after prolonged load carriage is unknown. In addition, as far as we known, a comparison of carbohydrate vs protein supplement on recovery of muscle function after prolonged load carriage has not been investigated.

The aim of this study was to compare the effects of commercially available carbohydrate vs whey protein supplements on recovery of muscle function after 2 hrs of treadmill walking (6.5 km·h^-1^) carrying a 25 kg backpack.

## Methods

### Participants

Ten healthy male participants (age 28 ± 9 years, height 1.82 ± 0.07 m, body mass 81.5 ± 10.5, body fat 16.4 ± 3.2%,  O_2_max 55.0 ± 5.5 ml·kg^-1^·min^-1^) volunteered for the study. Participants were all recreationally active and had a mixture of recreational and occupational experience of carrying load in backpacks. Ethical approval for procedures and protocols was provided by the University of Chichester Ethics Committee. All protocols were performed in accordance with the ethical standards laid down in the 2004 Declaration of Helsinki. Participants provided written informed consent and were free from musculoskeletal injury. Participants were not engaged in formal training with the muscle groups of interest. In the day prior and after load carriage, participants refrained from vigorous physical activity. On the day of load carriage, participants consumed a standardised light meal and avoided consumption of caffeine, sports drinks or food three hours prior to exercise. In the days after load carriage participants maintained their normal diet (recorded in a food diary, described in detail below) that was kept constant between test conditions. All testing was completed within a period of 5.9 ± 4.1 weeks.

### Preliminary Measures

Body mass (Seca Model 880, Seca Ltd., Birmingham, UK) was measured whilst wearing shorts and underwear. Skinfold measurements were taken at the *Biceps*, *Triceps*, *Sub Scapular *and *Iliac Crest *on the right side of the body using Harpenden Skinfold Callipers (Body Care, Southam, UK). Two measurements were taken at each site and if there was a difference > 10% the measurements were repeated. Percentage body fat was estimated following the assessment of skinfold thickness at the four anatomical sites.

At least 5 days prior to beginning the experimental protocol, participants were familiarised with all test procedures. Participants completed 3 maximal voluntary isometric contractions and all electrically stimulation procedures (described in detail below). The currents required to stimulate a maximal twitch force (group mean ± SD; 830 ± 67 mA) and sub-maximal twitch force (5% MVC force) (group mean ± SD; 420 ± 77 mA) were recorded and kept constant in all subsequent test sessions. Participants also completed 1 cycle of the isokinetic experimental protocol (described in detail below). A test procedure was repeated if the experimenter or participant thought that a maximal effort was not given or a learning effect was still apparent in the final contractions.

### Experimental Protocol

The study was a repeated measures three way cross over randomised design. There was a recovery period of at least two weeks between each experimental condition. All testing was performed at a laboratory temperature of about 21°. Participants walked for 2 hours at 6.5 km·h^-1 ^and 0% gradient carrying a 25 kg backpack on a motorised treadmill (Woodway Ergo ELG 70, Cranlea & Co, Birmingham, UK) [11]. The load was evenly distributed in the backpack. The backpack had adjustable shoulder straps, a fixed height waist strap that could be tightened but no sternum strap. Subjects adjusted the strapping to achieve a comfortable fit. Walking speed and absolute load reflects realistic occupational requirements (e.g. military load carriage). During each of the 3 testing sessions, participants consumed 500 ml of a commercially available beverage (250 ml at the start and 250 ml at 60 minutes of walking) mixed as directed by manufacturers guidelines:

(1) Placebo (PLA): 490 ml water and 10 ml sugar free orange cordial (Tesco, Dundee, UK), (nutritional content per 500 ml; Energy 1 Kcal, Carbohydrate 0.1 g, Protein 0 g, Fat 0 g)

(2) Carbohydrate (CHO) (6.4% carbohydrate concentration): 490 ml water, 10 ml sugar free orange cordial (Tesco, Dundee, UK), 34 g Super Soluble Maxijul (SHS International Limited, Liverpool, UK) (nutritional content per 500 ml; Energy 130 Kcal, Carbohydrate (100% glucose) 32 g, Protein 0 g, Fat 0 g),

(3) Protein (PRO) (7.0% protein concentration): 500 ml water and 44 g orange flavoured Maximuscle Promax (Maximuscle Limited, Hemel Hempstead, UK) (nutritional content per 500 ml; Energy 176 Kcal, Carbohydrate 3 g Protein 36 g, Fat 3 g, Sodium 0.2 g).

Participants consumed also 200 ml of water at 30 minutes and 200 ml water at 90 minutes of walking. Immediately after and in the evening after (~1900 hrs) each muscle testing session (described below), participants consumed 500 ml the allocated supplement (i.e. PLA, PRO or CHO). Absolute volumes of the supplement were provided to maintain ecological validity of consuming commercially available supplements. Participants were blind as to which supplement they were consuming.

Participants completed the muscle testing protocol before commencing load carriage (pre-exercise) and at 0 (immediately post), 24, 48 and 72 hours after load carriage. The test order was the same on each occasion and was conducted at approximately the same time of day. Three minutes rest was provided between each of the test procedures. During the recovery periods, participants refrained from any vigorous physical activity but outside of the testing periods (i.e. between experimental trials), they maintained their usual physical activity with none involved in specific training programs to improve physical fitness.

For isometric contractions of the knee extensors, participants were secured in a custom built chair with hip and knee at 90° flexion. Velcro straps around the participant's chest and waist restricted movement of upper body and hips. A cuff was placed around the participant's ankle and attached to an s-beam load cell (RS 250 kg, Tedea Huntleigh, Cardiff, UK) via a steel chain at the base of the chair. Muscle force was recorded on a computer at 1000 Hz using Chart 4 V4.1.2 (AD Instruments, Oxford, UK). Two custom made saline soaked electrodes (9 × 18 cm) were placed just above the patella and over the muscle belly of the knee extensors in the proximal third part of the thigh of the non-dominant leg. The position of the electrodes was marked using permanent pen to ensure accurate placement on subsequent tests. For all electrically evoked test procedures, stimulation was provided through an electrical muscle stimulator (Model DS7A, Digitimer Limited, Welwyn Garden City, UK) and pulses were controlled by a NeuroLog pulse generator (Digitimer Limited, Welwyn Garden City, UK). Participants conducted three 5 second sub-maximal contractions (~200 N) each testing session to become accustomed to the experimental set up.

### Isometric Maximal Voluntary Contraction (MVC)

Participants produced a 3 to 5 second maximal voluntary contraction (MVC) with strong verbal encouragement. When the effort was not considered maximal the procedure was repeated after 2 minutes rest. Approximately 90% of MVC's were maximal effort on the first attempt. The maximal force was taken as the absolute highest value during the contraction.

### Interpolated Doublet (% Voluntary Activation) During Isometric Contraction

A doublet pulse (two maximal single twitches separated by 10 ms) was applied to the knee extensors during the plateau phase of the MVC contraction, and immediately after the MVC when participants returned to rest (potentiated doublet). Percent voluntary activation (%VA) was calculated (Equation 1). The following parameters were calculated for the potentiated doublet: (a) peak force (N), the maximal force value of the doublet; (b) contraction time (s), the time between the first derivation from baseline and peak force; (c) average rate of force development (N·s^-1^), peak force/contraction time; (d) half relaxation time (s), the time taken to fall from peak force to half of the value during the relaxation phase; (e) maximal rate of force development (N·s^-1^), the highest value of the first derivative of the force signal; and (f) maximal rate of force decrease (N·s^-1^), the lowest value of the first derivative of the force signal.(1)

### Isometric 20 Hz and 50 Hz stimulation

20 Hz and 50 Hz stimulations (0.5 s duration), with 30 second rest between stimulations, were applied to the knee extensors using the sub-maximal twitch current (group mean ± SD; 420 ± 77 mA). A sub-maximal current gives a reliable estimate of contractile properties and is more tolerable for participants. A ratio of the forces at 20 Hz and 50 Hz was calculated, a reduction in the ratio indicates the presence of low frequency fatigue.

### Isokinetic Contractions

Three sub-maximal (self-perceived 50% effort) contractions (full extension and flexion) were completed to familiarise participants for each test. During all isokinetic tests, encouragement was standardised and participants were informed when they were half way through the test and had one repetition of the test remaining. Fast and slow isokinetic velocities were chosen as there are known variations in motor unit recruitment patterns and muscle fibre composition between individuals and between muscle groups [12].

Knee and shoulder extension and flexion data were recorded using HUMan Assessment Computer (HUMAC) software V40 (Computer Sports Medicine Inc, Norwood, USA) at 100 Hz. Data were corrected for the effect of gravity. Trunk extension and flexion data were recorded at 100 Hz using Akron software V2.4 (Akron Therapy Products, Ipswich, UK). Data were not corrected for the effect of gravity due to the limitations of the dynamometer, but changes over time can still be measured. Slower test velocities were tested first to increase reproducibility of results between tests [13]. Angular velocity was calculated every 0.01 seconds during the movement and data were removed if they were not collected during the isokinetic phase of the movement or showed torque overshoot [12]. Peak torque for each speed was taken as the maximum torque value of all contractions.

### Isokinetic Knee Extension and Flexion

Participants were seated (Cybex II isokinetic dynamometer, Cybex, Measham, UK) with knee secured at 90° flexion using a seat belt style strap across chest and hips. The Cybex long input adapter, adjustable arm and shin pad were attached to the dynamometers point of rotation and to the ankle of the non-dominant leg via a Velcro cuff. The dominant leg was behind the restraining bar to prevent movement. The point of rotation of the dynamometer arm was aligned with the *lateral femoral epicondyle *[14]. Participant range of motion was restricted by mechanical stops at 70° (flexion) and 0° (extension) of the knee. The protocol consisted of 2 sets of 5 maximal dynamic contractions of knee extensors and flexors at 60 and 180°·s^-1^, each separated by 30 s rest.

### Isokinetic Trunk Extension and Flexion

Participants were positioned standing upright (trunk fully extended, 0°) in an isokinetic trunk strength dynamometer (Akron Therapy Products, Ipswich, UK). Movement was restricted to use of the abdominal and back muscles between extension (5°) and flexion (50°) of the start position. Straps were placed across the participants upper and lower legs and hips and a frame positioned around the shoulders. The point of rotation of the dynamometer was aligned with the L5-S1 vertebrae [14]. The protocol consisted of 2 sets of 3 maximal dynamic contractions of the trunk extensors and flexors at 15 and 60°·s^-1^, each separated by 30 s rest.

### Isokinetic Shoulder Extension and Flexion

Participants lay in a supine position on a custom made testing couch placed parallel to a Cybex II isokinetic dynamometer (Cybex, Measham, UK). The Cybex offset input adapter, shoulder testing accessory and neutral handgrip were attached to the dynamometer. Participants griped the handle in their right hand; the adapter length was adjusted so their right arm was fully extended (0°) (i.e. minimal flexion in the elbow). Participants' movement was restricted by securing Velcro straps across the upper legs and hips with the left arm placed across the chest. The point of rotation of the dynamometer arm was aligned with the right *Acromiale *[14]. Participants were tested on their right arm only, but very little difference in strength exists between dominant and non- dominant arms [12]. Range of motion was between 0° and 180°. The test protocol consisted of 2 sets of 5 maximal dynamic contractions of the shoulder extensors and flexors at 60 and 180°·s^-1^, each separated by 30 s rest.

### Food Diary

Participants were instructed to consume a light meal (cereal and toast) at least 3 hours prior to treadmill walking sessions (PLA: 266 ± 157 Kcal (carbohydrate: 51 ± 37; fat 3 ± 3; protein: 11 ± 6), CHO: 259 ± 154 Kcal (carbohydrate: 49 ± 36; fat 3 ± 3; protein: 11 ± 6), PRO (277 ± 147 Kcal (carbohydrate: 55 ± 34; fat 3 ± 3; protein: 10 ± 6). There were no differences in macronutrient intake prior to treadmill walking between conditions (*P *> 0.05). Participants recorded any food or beverages (with estimated mass or portion size) consumed on the day of and for 72 hours after treadmill walking. Food diaries were analysed using Microdiet Plus for Windows V1.2 (Downlee Systems Ltd, Derbyshire, UK). There were no differences between conditions before or after load carriage in dietary intake of energy (Table 1).

**Table 1:** Dietary intake of energy, carbohydrate, fat and protein

Variable	Condition	24 h	48 h	72 h
Energy (Kcal)	PLA	1494 ± 740	1484 ± 659	1600 ± 549
	CHO	1547 ± 702	1468 ± 680	1532 ± 628
	PRO	1611 ± 658	1481 ± 626	1613 ± 534

Carbohydrate (g)	PLA	212 ± 162	217 ± 159	221 ± 108
	CHO	224 ± 156	209 ± 162	207 ± 111
	PRO	233 ± 150	216 ± 161	226 ± 106

Fat (g)	PLA	41 ± 24	41 ± 28	52 ± 28
	CHO	45 ± 28	45 ± 32	50 ± 26
	PRO	46 ± 27	43 ± 23	53 ± 23

Protein (g)	PLA	82 ± 26	73 ± 27	76 ± 21
	CHO	77 ± 22	69 ± 23	75 ± 22
	PRO	80 ± 23	69 ± 19	73 ± 21

Measured by food diaries after (24, 48 and 72 h) 120 minutes of treadmill walking at 6.5 km·h^-1 ^(n = 10) on a level gradient (0%) carrying a 25 kg backpack. Either a placebo beverage (PLA), carbohydrate (6.4%) beverage (CHO) or protein (7%) beverage (PRO) was consumed at 0 and 60 minutes (250 ml) during treadmill walking or twice daily (500 ml, morning and evening) for the 3 days after load carriage (n = 10). Data are presented excluding the consumption of the supplement beverages.

### Statistical Analysis

Statistical analysis was undertaken using SPSS for Windows V15 (SPSS, Chicago, Illinois). Normal distribution of the data was verified using a Kolmogorov-Smirnov test. Differences between groups and over time were assessed using 2 way repeated measures ANOVA. If sphericity was violated, the Greenhouse-Geisser correction was used. If the ANOVA revealed a significant interaction effect, differences were examined using pre-planned paired t-tests; over time (i.e. baseline vs. 0, 24, 48 or 72 h) or between conditions at each time point. The results are presented as mean ± standard deviation (SD). Statistical significance was set *a priori *at *P *< 0.05.

## Results

There were no differences in pre-exercise values for muscle force or torque of a specific muscle group between conditions suggesting the absence of muscle fatigue and/or injury before each bout of load carriage.

### Voluntary and Electrically Stimulated Isometric Contractions of the Knee Extensors

**Figure 1:**
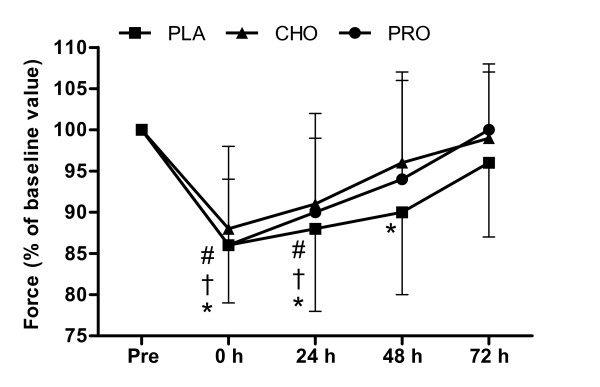
**Force of the knee extensors during isometric MVC**. Measurements were made before and after (0, 24, 48 and 72 h) 120 minutes of treadmill walking at 6.5 km·h^-1 ^(n = 10) on a level gradient (0%) carrying a 25 kg backpack with consumption of 250 ml (at 0 and 60 minutes) of a beverage containing placebo (PLA - Black square), carbohydrate (6.4%) (CHO - Black triangle) or protein (7%) (PRO - Black circle) and twice daily (500 ml, morning and evening) for the 3 days after load carriage (n = 10). Symbols show difference from pre measurement for PLA (* *P *< 0.05), CHO († *P *< 0.05), PRO (# *P *< 0.05).

The change in isometric force of knee extensors over time following load carriage was different between conditions (*P *< 0.001). Force decreased from pre-exercise value immediately after load carriage for PLA (14 ± 7%, *P *< 0.001), CHO (12 ± 10%, *P *= 0.006) and PRO (14 ± 8%, *P *< 0.001), with no difference between conditions (*P *> 0.05). At 24 h, isometric force was still below pre-exercise value for PLA (12 ± 10%, *P *= 0.009), CHO (9 ± 11%, *P *= 0.021) and PRO (10 ± 9%, *P *= 0.003). By 48 h, isometric force was 10 ± 10% below pre-exercise value for PLA (*P *= 0.008), but had returned to pre-exercise value for CHO (*P *= 0.199) and PRO (*P *= 0.099), respectively. At 72 h, PLA returned to pre-exercise value (*P *= 0.145) and both CHO (*P *= 0.457) and PRO (*P *= 0.731) remained at the pre-exercise value (Figure 1).

Voluntary activation changed over time (*P *= 0.016) but there was no difference between conditions (*P *= 0.848). VA decreased immediately after load carriage in all conditions (*P *= 0.034), but then recovered at 24 h (*P *= 0.086) and was not different from pre-exercise values at 48 (*P *= 0.067) and 72 h (*P *= 0.243) (Additional file 1).

The 20:50 Hz force ratio was lower before exercise for PRO compared to PLA (*P *= 0.030) and CHO (*P *= 0.019), but there was no difference between CHO and PLA (*P *= 0.795) (Additional file 1). The 20:50 Hz force ratio changed over time (*P *= 0.027) but there was no difference between conditions (*P *= 0.257). Immediately after load carriage there was no change in the 20:50 Hz force ratio (*P *= 0.100). The 20:50 Hz force ratio was lower than the pre-exercise value at 24 h (*P *= 0.031) and 48 h (*P *= 0.018), returning to the pre-exercise value at 72 h (*P *= 0.443) (Additional file 1).

Doublet contraction time changed over time (*P *= 0.029) but there was no difference between conditions (*P *= 0.845). Contraction time decreased immediately after load carriage in all conditions (*P *= 0.032) and returned and remained above pre-exercise value at 24, 48 and 72 h (*P *> 0.05) (Additional file 1).

Doublet half relaxation time changed over time (*P *= 0.038) with no differences between conditions (*P *= 0.643). Half relaxation time decreased immediately after exercise (*P *< 0.05), and made a temporary recovery at 24 h, then decreased below pre exercise value at 48 h (*P *< 0.05), returning to pre-exercise values again at 72 h (*P *= 0.251) (Additional file 1).

Additional file 1 shows that there were no changes over time in any condition for the doublet peak force (*P *= 0.707), average rate of tension development (*P *= 0.497), maximal rate of force development (*P *= 0.380) or maximal rate of force decrease (*P *= 0.392).

### Isokinetic Contractions of the Knee Extensors

**Figure 2:**
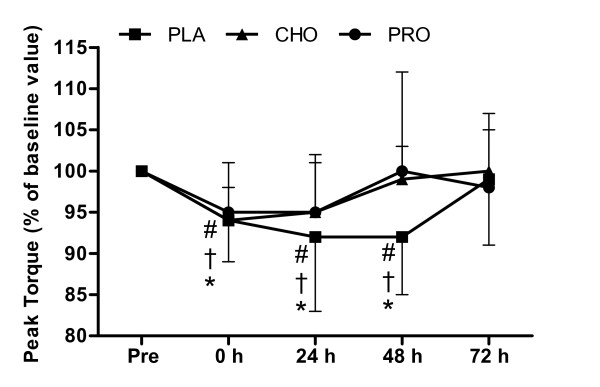
**Peak torque of the knee extensors during isokinetic contractions (60°·s^-1^)**. Measurements were made before and after (0, 24, 48 and 72 h) 120 minutes of treadmill walking at 6.5 km·h^-1 ^(n = 10) on a level gradient (0%) carrying a 25 kg backpack with consumption of 250 ml (at 0 and 60 minutes) of a beverage containing either placebo (PLA - Black square), carbohydrate (6.4%) (CHO - Black triangle) or protein (7%) (PRO - Black circle) and twice daily (500 ml, morning and evening) for the 3 days after load carriage (n = 10). Symbols show difference from pre measurement for PLA (* *P *< 0.05), CHO († *P *< 0.05), PRO (# *P *< 0.05).

Figure 2 shows that peak torque (60°·s^-1^) of knee extensors decreased immediately after load carriage (*P *< 0.001) remaining below pre-exercise value at 24 h (*P *= 0.001) and 48 h (*P *= 0.009) fully recovering at 72 h (*P *= 0.401). There was no difference between conditions (*P *= 0.242). There was no change over time in any condition for peak torque (180°·s^-1^) of the knee extensors (*P *= 0.053) (Table 2).

**Table 2:** Peak torque of the knee and trunk extensors and flexors during isokinetic contractions

Variable	Condition	Peak Torque (Nm)				
		
		Pre	0 h	24 h	48 h	72 h
Knee Extension	PLA	148 ± 20	145 ± 22	141 ± 28	149 ± 23	154 ± 24
(180 **°**·s^-1^)	CHO	150 ± 17	151 ± 16	152 ± 19	155 ± 21	147 ± 20
(n = 8)	PRO	150 ± 24	149 ± 23	140 ± 28	145 ± 24	146 ± 22

Knee Flexion	PLA	95 ± 21	85 ± 20 *	90 ± 28	97 ± 21	102 ± 18
(180 **°**·s^-1^)	CHO	97 ± 9	86 ± 17 *	96 ± 21	95 ± 17	92 ± 16
(n = 8)	PRO	98 ± 13	90 ± 15*	96 ± 15	99 ± 19	97 ± 14

Trunk Extension	PLA	243 ± 55	218 ± 71 *	220 ± 57	244 ± 54	237 ± 56
(15 **°**·s^-1^)	CHO	259 ± 49	221 ± 45 *	236 ± 63	252 ± 60	256 ± 61
(n = 9)	PRO	242 ± 54	218 ± 62 *	243 ± 55	249 ± 73	241 ± 65

Trunk Extension	PLA	232 ± 56	204 ± 65 *	207 ± 55	227 ± 43	230 ± 60
(60 **°**·s^-1^)	CHO	226 ± 61	196 ± 51 *	221 ± 76	218 ± 72	243 ± 75
(n = 9)	PRO	232 ± 77	196 ± 60 *	229 ± 68	244 ± 62	233 ± 81

Trunk Flexion	PLA	298 ± 36	275 ± 34 *	298 ± 28	296 ± 44	310 ± 25
(60 **°**·s^-1^)	CHO	300 ± 36	284 ± 40 *	301 ± 35	300 ± 32	299 ± 40
(n = 10)	PRO	299 ± 36	277 ± 38 *	289 ± 43	296 ± 44	299 ± 34

Measurements were taken before (Pre) and after (0, 24, 48 and 72 h) 120 minutes of treadmill walking at 6.5 km·h^-1 ^(n = 10) on a level gradient (0%) carrying a 25 kg backpack. Either a placebo beverage (PLA), carbohydrate (6.4%) beverage (CHO) or protein (7%) beverage (PRO) was consumed at 0 and 60 minutes (250 ml) during treadmill walking or twice daily (500 ml, morning and evening) for the 3 days following load carriage. *, different from pre-value (P < 0.05).

### Isokinetic Contractions of the Knee Flexors

**Figure 3:**
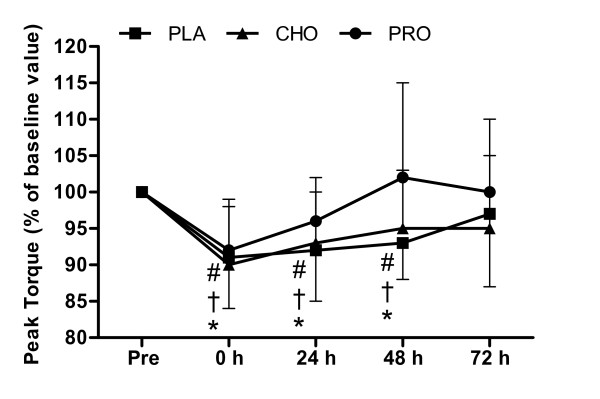
**Peak torque of the knee flexors during isokinetic contractions (60°·s^-1^) **Measurements were made before and after (0, 24, 48 and 72 h) 120 minutes of treadmill walking at 6.5 km·h^-1 ^(n = 10) on a level gradient (0%) carrying a 25 kg backpack with consumption of 250 ml (at 0 and 60 minutes) of a beverage containing either placebo (PLA - Black square), carbohydrate (6.4%) (CHO - Black triangle) or protein (7%) (PRO - Black circle) and twice daily (500 ml, morning and evening) for the 3 days after load carriage (n = 10). Symbols show difference from pre measurement for PLA (* *P *< 0.05), CHO († *P *< 0.05), PRO (# *P *< 0.05).

Peak torque (60°·s^-1^) of knee flexors changed over time (*P *< 0.001) but there was no difference between conditions (*P *= 0.762) (Figure 3). Knee flexor peak torque (60°·s^-1^) decreased below pre-exercise value (*P *< 0.001) and remained suppressed at 24 h (*P *= 0.001) and 48 h (*P *= 0.012) fully recovering by 72 h (*P *= 0.109). Knee flexor peak torque (180°·s^-1^) decreased immediately after load carriage in all conditions (*P *= 0.010) and fully recovered 24 h (*P *= 0.397) remaining at pre-exercise value for all conditions at 48 and 72 h (P > 0.05). There was no difference between conditions (*P *= 0.481).

### Isokinetic Contractions of the Trunk Extensors

Peak torque (15°·s^-1^) of the trunk extensors decreased immediately after load carriage in all conditions (*P *< 0.001), and recovered at 24 h (*P *= 0.091) remaining above pre-exercise values at 48 and 72 h (P > 0.05). There was no difference between conditions (*P *= 0.680). Similarly, peak torque (60°·s^-1^) of the trunk extensors decreased immediately after load carriage in all conditions (*P *< 0.020), and recovered at 24 h (*P *= 0.058) remaining above pre-exercise values at 48 and 72 h (P > 0.05) There was no difference between conditions (*P *= 0.461) (Table 2).

### Isokinetic Contractions of the Trunk Flexors

**Figure 4:**
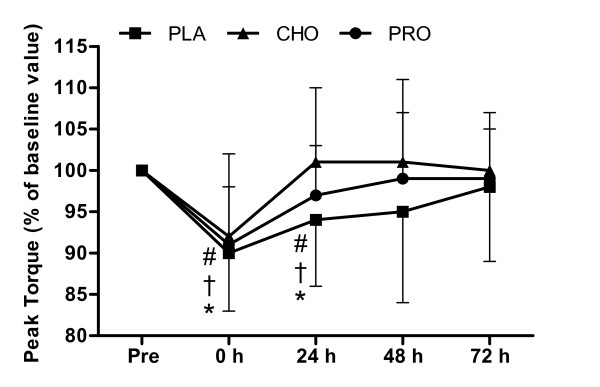
**Peak torque of the trunk flexors during isokinetic contractions (15°·s^-1^)**. Measurements were made before and after (0, 24, 48 and 72 h) 120 minutes of treadmill walking at 6.5 km·h^-1 ^(n = 10) on a level gradient (0%) carrying a 25 kg backpack with consumption of 250 ml (at 0 and 60 minutes) of a beverage containing either placebo (PLA - Black square), carbohydrate (6.4%) (CHO - Black triangle) or protein (7%) (PRO - Black circle) and twice daily (500 ml, morning and evening) for the 3 days after load carriage (n = 10). Symbols show difference from pre measurement for PLA (* *P *< 0.05), CHO († *P *< 0.05), PRO (# *P *< 0.05).

Figure 4 shows that peak torque (15°·s^-1^) of the trunk flexors decreased immediately after load carriage in all conditions (*P *< 0.001) and remained below pre-exercise value at 24 h (P = 0.019) and was fully recovered at 48 and 72 h (P > 0.05). There were no differences between conditions (*P *= 0.768). Peak torque (60°·s^-1^) of the trunk flexors decreased immediately after load carriage in all conditions (*P *= 0.005) returning and remaining above pre-exercise value at 24, 48 and 72 h (*P *> 0.05). There was no difference between conditions (*P *= 0.662).

### Isokinetic Contractions of the Shoulder Extensors and Flexors

There were no changes over time in any condition for the shoulder extensors (60°·s^-1^) (*P *= 0.124), shoulder extensors (180°·s^-1^) (*P *= 0.101), shoulder flexors (60°·s^-1^) (*P *= 0.094) or shoulder flexors (180°·s^-1^) (*P *= 0.078).

### Discussion

The primary finding of the present study was that time course of recovery of neuromuscular function following prolonged load carriage (2 h, 25 kg) is improved with consumption of whey protein and carbohydrate beverages. After load carriage, isometric knee extension force recovered to pre-exercise values following 48 h recovery with carbohydrate and whey protein beverages compared to 72 h recovery with a placebo. Interestingly, recovery of isokinetic peak torque was not improved by supplementation. However, our experimental model had similar absolute loads during load carriage that may have resulted in large variation. It is possible that this large variation and our choice of analysing different recovery time points has masked, for example, potential improved effects of both supplements at 48 h for peak torque (60°·s^-1^) of the knee extensors (Figure 2) and the effect of whey protein at 48 h for peak torque (60°·s^-1^) of knee flexors (Figure 3).

Reductions in torque in the present study are supported by data of Clarke *et al. *[1], which showed decreases in strength of knee and trunk extensors and flexors after a 12.1 km road march at 4 km·h^-1 ^carrying a 27 kg load. Clarke *et al. *[1] observed larger decreases in knee extensor peak torque (6 vs. 8%) but smaller decreases in knee flexor peak torque (9 vs. 6%) with comparable reductions for changes in trunk extensor (12 vs. 11%) and flexor peak torque (10 vs. 11%).

Whey protein intake during resistance training has been shown to improve muscle hypertrophy [7] and maintain a positive protein balance [15]. The effect of whey protein supplementation on recovery of muscle function after resistance or endurance exercise has received little attention. Buckley *et al. *[16] observed a ~23% decrease in isometric force of the knee extensors after 100 maximal eccentric contractions. During 24 h recovery, isometric force did not recover to pre-exercise values with placebo, but consumption of 25 g of whey protein hydrolysate immediately after exercise resulted in complete recovery of isometric MVC force by 6 h post-exercise [16].

It has been shown that protein supplementation during and after exercise promotes and provides building blocks for *de novo *protein synthesis and reduces protein degradation, ensuring a positive protein balance [17]. Such maintenance of an anabolic rather than catabolic environment will enhance muscle protein accretion [18], probably resulting in enhanced repair of the structural muscle proteins damaged during exercise. Indeed, Nosaka [19] suggested the greater rate of protein synthesis and reduced protein breakdown when amino acids are ingested will reduce the magnitude of muscle damage and improve the rate of recovery. This may explain faster recovery for isometric knee extensor peak force with the whey protein beverage compared to placebo.

The data in the present study show that carbohydrate supplementation during load carriage does not effect force of the knee extensors immediately after load carriage. However, compared to placebo, carbohydrate showed beneficial effects in promoting faster recovery of muscle function. In contrast to these findings, Nelson *et al. *[20], showed no effect on the recovery of muscle function after a 15 minute downhill run in a glycogen depleted state when a high carbohydrate diet (80% carbohydrate) was consumed compared to no food. However, Nelson *et al. *[20] provided only a single high carbohydrate meal immediately after exercise with no dietary control afterwards. In the present study, carbohydrate beverages were consumed twice daily during recovery and there were no differences in macronutrient intake. During prolonged exercise muscle glycogen stores have been shown to be reduced [21] and fatigue coincides with depleted muscle glycogen stores. Glycogen depleted fibres exhibit higher energy deficiency due to elevated post exercise inosine 5'-monophosphate (IMP) concentrations (a marker of the mismatch between ATP re-synthesis and degradation) [22]. Although these data suggest compromised muscle function by glycogen depletion, there is no experimental evidence from in vivo studies linking muscle glycogen concentration and performance during short-duration isometric or isokinetic contractions.

The extent to which the carbohydrate supplements in the present study enhanced muscle glycogen stores is debatable as the effect in sparing muscle or liver glycogen stores appears to be dependent on exercise mode, intensity and duration. The provision of carbohydrate supplements after exercise has been shown to improve glycogen synthesis [10]. However, in the present study 500 ml of the 6.4% carbohydrate supplement was consumed twice daily in one bolus, providing 32 g of carbohydrate (~0.3 g·kg body mass^-1^·h^-1 ^in the hour after exercise), which is considerably less than the 1.2 g·kg body mass^-1^·h^-1 ^believed to be optimal for restoration of muscle glycogen [23]. Also, the benefits of exogenous carbohydrate supplementation to restore muscle glycogen appear to occur in the first 4-6 hours after an exercise bout [10,15]. Therefore, the effects are likely to have only been observed up to 24 h after load carriage in the present study.

The preceding discussion suggests that carbohydrate supplementation in the present study had a minimal effect in improving muscle glycogen concentration and if so it is unlikely to account for the improved recovery of muscle function. The carbohydrate supplement would have increased blood glucose and insulin release. Insulin increases the rate of protein synthesis at rest and attenuates the rate of protein breakdown after exercise [24]. Therefore, carbohydrate may have decreased the negative protein balance after exercise compared to placebo, slowing the degradation of structural proteins with a positive effect on recovery of muscle function.

Beverages were consumed in the three days following load carriage, immediately after each muscle testing session and each evening. Ingestion of additional PRO and CHO between meals may have provided a more consistent supply of macronutrients and increased insulin concentrations compared to PLA (when nutrients were only consumed during meal times). PRO supplementation provided amino acids and promoted insulin release, but it is likely that the insulin response would have been higher with CHO supplementation compared to PRO. Thus, both supplementation strategies reduce the negative protein balance through different mechanisms. However, there did not appear to be a difference between PRO and CHO supplementation on neuromuscular function in our study. However, the precise effect of PRO and CHO supplementation is rather speculative as exact timings of participants meals were not recorded, but participant food diaries indicate that eating habits were similar between conditions.

In our study, whey protein and carbohydrate supplements had no effect on the recovery of the 20:50 Hz force ratio, contraction and relaxation times. The faster contraction and half relaxation times immediately after load carriage were surprising as fatigued muscles generally show a slowing of contraction and relaxation velocity [25]. However, the changes in the contraction and relaxation time following exercise due to neuromuscular impairment (i.e. a slowing) may have been masked by potentiation, which increases the speed of contraction and half relaxation times [26].

Voluntary activation decreased immediately after load carriage and remained above pre-exercise value from 24 h onwards during recovery in all conditions (Additional file 1). This indicates part of the neuromuscular impairment immediately after exercise could be accounted for by central mechanisms [25] but the supplements had no effect on this response. This is surprising as it has been suggested that branched chain amino acids (BCAA) are a beneficial nutrient in delaying the onset of central fatigue as they compete with tryptophan for transport into the brain and consequently reduce brain serotonin [27]. BCAA constitute approximately 23% of the whey protein supplement. However, during sustained exercise BCAAs are also taken up by the muscle and the plasma concentration decreases, potentially giving rise to more tryptophan crossing the blood brain barrier. Whey proteins also contain between 20-25% of alpha-lactalbumin, ingestion of which has been indirectly shown to increase brain serotonin activity [28]. Thus, the net effect of the ingestion of a large bolus (33 g) of whey protein during endurance exercise may actually increase brain serotonin activity and hastens central fatigue [29].

Interestingly, peak torque during isokinetic contractions in all muscle groups showed no difference in the pattern of recovery which is in contrast to the differences discussed previously for maximal force of the isometric contractions. However, we have no clear explanation what may explain the difference but it could be related to differences in cross-bridge action during isokinetic versus isometric contractions.

Compared to most recent Institute of Medicine recommendations [30], the data in Table 1 suggested that during the 72 h after the load carriage bout the participants in the present study were approximately in deficit of 1173 Kcal·day^-1 ^energy, 129 g·day^-1 ^carbohydrate and 37 g·day^-1 ^fat, but participants did consume 16 g·day^-1 ^protein above recommended guidelines. However, it has been shown that self report food diaries consistently underreport nutritional intake [31]. Participants maintained their normal dietary intake throughout the study and were weighed prior to each load carriage bout, the number of days between their first and last test was 41 ± 29 days. Assuming surplus energy is stored on a fat:fat free mass ratio of 75:25, a change in body mass of 1 kg can be assumed to be equivalent of ~7170 kcal [32]. If the participants had been in negative energy balance of ~1173 Kcal (as the food diaries indicate) for ~41 days (time between first and last body mass measurement) participants would have lost an average ~6.7 kg. However, there was no difference in body mass between the first and last load carriage bout (82.0 ± 10.2 vs. 82.0 ± 10.7 kg, *P *= 0.990). These findings suggest participants were not in negative energy balance and therefore not in nutritional deficit during the recovery period. However, we did not standardize the characteristics of physical activity allowed by subjects between the three testing sessions including the recovery period.

There were no differences in dietary intake of energy, carbohydrate, fat or protein over the 72 h that recovery of muscle function was measured after load carriage. Compared to the placebo the carbohydrate and whey protein beverages provided an additional 260 Kcal and 352 Kcal·day^-1^, respectively. However, Valentine *et al. *[33] showed that the calorific content of a sports drink has no effect on indices of muscle disruption (creatine kinase concentration), but differences were shown between calorie matched carbohydrate and carbohydrate and protein beverages. In addition, energy intake *per se *does not have an influence muscle protein metabolism after exercise, but may do if individuals were in chronic energy deficit [34]. These data indicate that it is the macronutrients content of the beverages that influence recovery of neuromuscular function following exercise rather than the calories *per se*.

In conclusion, prolonged load carriage resulted in similar reductions in isometric peak force of the knee extensors and isokinetic peak torque of the knee and trunk extensors and flexors and immediately after exercise, independent of the supplement consumed. Consumption of whey protein and carbohydrate supplements resulted in faster recovery of the isometric force of the knee extensors compared to a placebo. However, recovery of peak torque during isokinetic contractions in all muscle groups showed no difference in the pattern of recovery between conditions. We speculate that faster recovery of muscle function during isometric contractions after load carriage may have been due to the effect of carbohydrate and whey protein on protein synthesis and breakdown. Maintenance of an anabolic environment may have enhanced the repair of structural muscle proteins damaged during exercise leading to improved isometric muscle function during recovery from prolonged load carriage.

## Competing interests

The authors declare that they have no competing interests.

## Authors' contributions

SB, JB, JF and MW all contributed to the study design. SB and NW recruited participants and conducted all data collection. SB undertook analysis of all the data. SB and MW both interpreted the data. All authors reviewed and approved the final manuscript.

## Supplementary Material

Additional file 1 **Responses during electrically stimulated isometric contractions of the knee extensors**. Table with measurements that were taken before (Pre) and after (0, 24, 48 and 72 h) 120 minutes of treadmill walking at 6.5 km·h^-1 ^(n = 10) on a level gradient (0%) carrying a 25 kg backpack. Either a placebo beverage (PLA), carbohydrate (6.4%) beverage (CHO) or protein (7%) beverage (PRO) was consumed at 0 and 60 minutes (250 ml) during treadmill walking or twice daily (500 ml, morning and evening) for the 3 days after load carriage (n = 10). *, different from pre-value (P < 0.05). Click here for file
