# Supplementary material for: Carbohydrate vs protein supplementation for recovery of neuromuscular function following prolonged load carriage
Source: J Int Soc Sports Nutr. 2010 Jan 12;7:2. doi: 10.1186/1550-2783-7-2 (PMC2821364; doi:10.1186/1550-2783-7-2)
Supplement: Additional file 1 — Responses during electrically stimulated isometric contractions of the knee extensors. Table with measurements that were taken before (Pre) and after (0, 24, 48 and 72 h) 120 minutes of treadmill walking at 6.5 km·h-1 (n = 10) on a level gradient (0%) carrying a 25 kg backpack. Either a placebo beverage (PLA), carbohydrate (6.4%) beverage (CHO) or protein (7%) beverage (PRO) was consumed at 0 and 60 minutes (250 ml) during treadmill walking or twice daily (500 ml, morning and evening) for the 3 days after load carriage (n = 10). *, different from pre-value (P < 0.05). [file 1550-2783-7-2-S1.DOC]

**Responses during electrically stimulated isometric contractions of the knee extensors*.*** Measurements were taken before (Pre) and after (0, 24, 48 and 72 h) 120 minutes of treadmill walking at 6.5 km·h^-1^ (n=10) on a level gradient (0%) carrying a 25 kg backpack. Either a placebo beverage (PLA), carbohydrate (6.4%) beverage (CHO) or protein (7%) beverage (PRO) was consumed at 0 and 60 minutes (250 ml) during treadmill walking or twice daily (500 ml, morning and evening) for the 3 days after load carriage (n = 10). *, different from pre-value (P<0.05).

| Variable | Condition | Pre | | | 0 h | | | 24 h | | | 48 h | | | 72 h | | |
| --- | --- | --- | --- | --- | --- | --- | --- | --- | --- | --- | --- | --- | --- | --- | --- | --- |
| VA (%) | PLA | 98 | ± | 4 | 93 | ± | 11* | 96 | ± | 3 | 95 | ± | 9 | 96 | ± | 7 |
|  | CHO | 97 | ± | 4 | 92 | ± | 9* | 95 | ± | 9 | 95 | ± | 5 | 97 | ± | 7 |
|  | PRO | 98 | ± | 3 | 92 | ± | 6 * | 93 | ± | 10 | 94 | ± | 9 | 96 | ± | 7 |
| Doublet Peak Force (N) | PLA | 174 | ± | 39 | 173 | ± | 39 | 170 | ± | 46 | 175 | ± | 50 | 176 | ± | 33 |
|  | CHO | 171 | ± | 41 | 166 | ± | 34 | 171 | ± | 32 | 170 | ± | 29 | 169 | ± | 34 |
|  | PRO | 175 | ± | 44 | 174 | ± | 46 | 171 | ± | 50 | 172 | ± | 42 | 174 | ± | 42 |
| Doublet Contraction Time (s) | PLA | 0.187 | ± | 0.007 | 0.181 | ± | 0.009* | 0.187 | ± | 0.010 | 0.187 | ± | 0.017 | 0.189 | ± | 0.011 |
|  | CHO | 0.185 | ± | 0.006 | 0.184 | ± | 0.010* | 0.187 | ± | 0.009 | 0.185 | ± | 0.009 | 0.187 | ± | 0.006 |
|  | PRO | 0.184 | ± | 0.011 | 0.182 | ± | 0.007* | 0.186 | ± | 0.010 | 0.186 | ± | 0.009 | 0.188 | ± | 0.008 |
| Average Rate of Doublet Tension Development (N·s^-1^) | PLA | 933 | ± | 214 | 953 | ± | 218 | 906 | ± | 237 | 930 | ± | 234 | 932 | ± | 163 |
|  | CHO | 922 | ± | 207 | 904 | ± | 179 | 915 | ± | 185 | 919 | ± | 168 | 901 | ± | 173 |
|  | PRO | 948 | ± | 227 | 951 | ± | 236 | 920 | ± | 249 | 921 | ± | 195 | 927 | ± | 220 |
| Doublet Half Relaxation Time (s) | PLA | 0.100 | ± | 0.009 | 0.098 | ± | 0.009* | 0.099 | ± | 0.011 | 0.099 | ± | 0.011* | 0.100 | ± | 0.012 |
|  | CHO | 0.100 | ± | 0.011 | 0.096 | ± | 0.006* | 0.100 | ± | 0.012 | 0.098 | ± | 0.010* | 0.097 | ± | 0.008 |
|  | PRO | 0.100 | ± | 0.013 | 0.095 | ± | 0.010* | 0.099 | ± | 0.011 | 0.098 | ± | 0.010* | 0.099 | ± | 0.009 |
| Doublet Maximal Rate of Force Development (N·s^-1^) | PLA | 1664 | ± | 402 | 1631 | ± | 379 | 1602 | ± | 406 | 1649 | ± | 412 | 1681 | ± | 310 |
|  | CHO | 1650 | ± | 406 | 1594 | ± | 318 | 1647 | ± | 356 | 1618 | ± | 328 | 1613 | ± | 318 |
|  | PRO | 1679 | ± | 422 | 1656 | ± | 427 | 1616 | ± | 410 | 1652 | ± | 365 | 1671 | ± | 359 |
| Doublet Maximal Rate of Force Decrease (N·s^-1^) | PLA | -1326 | ± | 354 | -1348 | ± | 371 | -1301 | ± | 390 | -1324 | ± | 389 | -1339 | ± | 335 |
|  | CHO | -1315 | ± | 362 | -1326 | ± | 294 | -1311 | ± | 372 | -1330 | ± | 292 | -1319 | ± | 304 |
|  | PRO | -1350 | ± | 407 | -1405 | ± | 420 | -1315 | ± | 397 | -1339 | ± | 383 | -1343 | ± | 344 |
| 20:50 Hz Ratio | PLA | 0.88 | ± | 0.04 | 0.83 | ± | 0.06 | 0.83 | ± | 0.07* | 0.83 | ± | 0.06* | 0.85 | ± | 0.06 |
|  | CHO | 0.87 | ± | 0.05 | 0.85 | ± | 0.06 | 0.83 | ± | 0.04* | 0.83 | ± | 0.03* | 0.85 | ± | 0.07 |
|  | PRO | 0.84 | ± | 0.06 ^†^ | 0.82 | ± | 0.07 | 0.81 | ± | 0.08* | 0.83 | ± | 0.05* | 0.86 | ± | 0.06 |
